# Natural history collections are critical resources for contemporary and future studies of urban evolution

**DOI:** 10.1111/eva.13045

**Published:** 2020-07-02

**Authors:** Allison J. Shultz, Benjamin J. Adams, Kayce C. Bell, William B. Ludt, Gregory B. Pauly, Jann E. Vendetti

**Affiliations:** ^1^ Urban Nature Research Center Natural History Museum of Los Angeles County Los Angeles CA USA; ^2^ Ornithology Department Natural History Museum of Los Angeles County Los Angeles CA USA; ^3^ Entomology Department Natural History Museum of Los Angeles County Los Angeles CA USA; ^4^ Department of Biological Sciences George Washington University Washington DC USA; ^5^ Mammalogy Department Natural History Museum of Los Angeles County Los Angeles CA USA; ^6^ Ichthyology Department Natural History Museum of Los Angeles County Los Angeles CA USA; ^7^ Herpetology Department Natural History Museum of Los Angeles County Los Angeles CA USA; ^8^ Malacology Department Natural History Museum of Los Angeles County Los Angeles CA USA

**Keywords:** biological evolution, museums, natural history, research methodology, trends, urbanization

## Abstract

Urban environments are among the fastest changing habitats on the planet, and this change has evolutionary implications for the organisms inhabiting them. Herein, we demonstrate that natural history collections are critical resources for urban evolution studies. The specimens housed in these collections provide great potential for diverse types of urban evolution research, and strategic deposition of specimens and other materials from contemporary studies will determine the resources and research questions available to future urban evolutionary biologists. As natural history collections are windows into the past, they provide a crucial historical timescale for urban evolution research. While the importance of museum collections for research is generally appreciated, their utility in the study of urban evolution has not been explicitly evaluated. Here, we: (a) demonstrate that museum collections can greatly enhance urban evolution studies, (b) review patterns of specimen use and deposition in the urban evolution literature, (c) analyze how urban versus rural and native versus nonnative vertebrate species are being deposited in museum collections, and (d) make recommendations to researchers, museum professionals, scientific journal editors, funding agencies, permitting agencies, and professional societies to improve archiving policies. Our analyses of recent urban evolution studies reveal that museum specimens can be used for diverse research questions, but they are used infrequently. Further, although nearly all studies we analyzed generated resources that could be deposited in natural history collections (e.g., collected specimens), a minority (12%) of studies actually did so. Depositing such resources in collections is crucial to allow the scientific community to verify, replicate, and/or re‐visit prior research. Therefore, to ensure that adequate museum resources are available for future urban evolutionary biology research, the research community—from practicing biologists to funding agencies and professional societies—must make adjustments that prioritize the collection and deposition of urban specimens.

## 1 INTRODUCTION

As the human population and the amount of urbanized habitat increase, associated environmental changes impact the organisms that inhabit urban areas (McDonald, 2008). These environmental changes produce not only ecological, but also evolutionary responses (Johnson & Munshi‐South, 2017). A growing number of studies examine evolutionary responses to urbanization (Johnson & Munshi‐South, 2017; Rivkin et al., 2019; Santangelo, Rivkin, & Johnson, 2018), most often by analyzing contemporary urban‐living populations, or by comparing urban populations to their rural‐living counterparts. While this research has produced important insights into how populations evolve in urban areas, ecologists recognize that results from analyses across space do not always replicate results from analyses through time (Johnson & Miyanishi, 2008; Wolkovich et al., 2012). This is also true for evolutionary biology, where temporal sampling can help disentangle the effects of multiple selective pressures, reveal signatures of selection obscured by demography, and facilitate clearer understandings of evolutionary processes (Habel, Husemann, Finger, Danley, & Zachos, 2013; Mathieson et al., 2015; Shultz, Baker, Hill, & Nolan, 2016). The specimens housed in natural history museums can provide this critical temporal study design.

For natural history museums and a wide range of biologists, specimen collecting is considered essential for understanding and preserving biodiversity, and chronicling its change through time (Allmon, 2005; Bakker et al., 2020; McLean et al., 2016; Rocha et al., 2014; Suarez & Tsutsui, 2004). New technologies, and their creative applications, have revolutionized the types of data that can be derived from natural history collection specimens (referred to as museum specimens hereafter) (Schindel & Cook, 2018). Many museum specimens have the added value of having been collected on historical timescales (e.g., ca. 1850–present for North America) and from changing landscapes and, therefore, are directly relevant for studying urban evolution (Lister & Climate Change Research Group, 2011). Given the importance of such existing collections, we advocate for increased scientific collecting in urban areas and additional support for such efforts, thereby enabling more contemporary and future investigations of urban‐associated evolutionary processes (Holmes et al., 2016), particularly during the Anthropocene (Meineke, Davies, Davies, Daru, & Davis, 2018; Schmitt, Cook, Zamudio, & Edwards, 2018). In this perspective paper, we (a) demonstrate the potential for museum specimens to greatly enhance studies of urban evolution via case studies; (b) quantify, from a literature search, how much museum specimens have been used in this context and how many studies archive or deposit resources they create (e.g., specimens); (c) analyze museum specimen deposition across four vertebrate groups and through space and time to better inform what existing specimens might be available for scientific study; and (d) advocate for specimen deposition and provide recommendations for stakeholders throughout the scientific community. With this information, we hope to increase the rate of specimen collection and deposition from urban environments to ensure that resources are being created for future urban evolution studies.

## 2 MUSEUM COLLECTIONS CAN GREATLY ENHANCE STUDIES OF URBAN EVOLUTION

**FIGURE 1:**
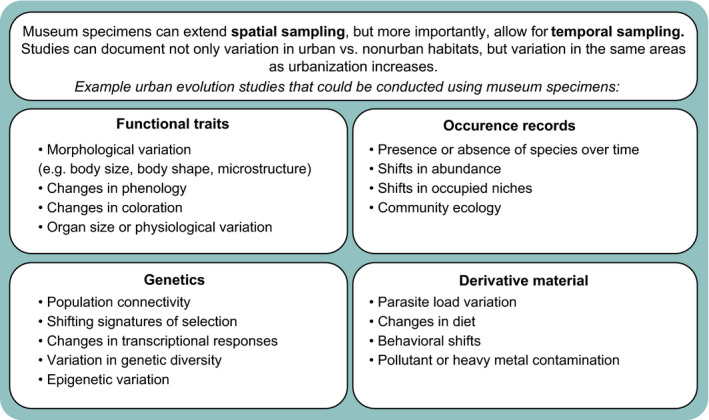
Example topics that could be studied using museum specimens to study urban evolution

Museum specimens uniquely allow researchers to examine change through time in morphology, distribution, genetic diversity, allele frequency, and life‐history traits (Figure 1). Below we provide examples of how museum specimens have been used in studies of urban evolution, and highlight opportunities for future work by including examples of innovative research based on museum specimens collected from nonurban habitats. We divide this section into four broad categories: (a) analysis/identification of functional traits, (b) recognizing changes in species distributions, (c) genetics, and (d) use of derivative materials (e.g., stomach contents, parasites, and photographs).

### 2.1 The evolution of functional traits

Museum specimens can provide rich and varied sources of data useful for studying the evolution of phenotypes and functional traits (e.g., assessed from morphology, phenology, and/or physiology) in organisms from urban environments. The vast majority of collections‐based studies focus on taxa from their undeveloped native habitat, while far fewer investigate phenotypic evolution temporally or spatially in urban‐living native or non‐native taxa. For example, in museum collections of native urban‐living mammals, cranial capacity (measured from skulls) was found to have increased in several taxa over time compared to the same taxa living in rural habitats (Snell‐Rood & Wick, 2013). In urban‐living fence lizards, limb and toe lengths were shorter compared to conspecifics from more natural areas based on measurements of museum specimens (Putman, Gasca, Blumstein, & Pauly, 2019). In stickleback fish and minnows, anthropogenic change to aquatic habitats resulted in varied phenotypic changes in body morphology (Cureton & Broughton, 2014; Kern & Langerhans, 2018; Kitano et al., 2008; Pease, Grabowski, Pease, & Bean, 2018). In urban‐living non‐native plants, changes in phenology, notably early onset of flowering, were linked to the influences of urban heat islands (Lavoie & Lachance, 2006; Neil, Landrum, & Wu, 2010).

With recent advances in technology applicable to museum specimens (e.g., CT‐scanning, super‐resolution microscopy, 3D tomography), morphological data that are invisible to the naked eye or hidden within the preserved organism may be visualized, bringing new research potential for museum specimens including those collected from urban environments (Gutiérrez, Ott, Töpperwien, Salditt, & Scherber, 2018; James, 2017).

### 2.2 Historical occurrence records provide a baseline for distributional studies

Museum vouchers document species occurrences in time and place. Museum specimens collected from urbanized regions are used as first occurrence records for documenting the presence and range of nonnative species, incorporated into regional landscape planning, and used as historical baselines for conservation policies and ecological analyses (Lister & Climate Change Research Group, 2011; McKinney, 2008; Sánchez‐Bayo & Wyckhuys, 2019). While diverse collection approaches and varied acquisition histories may result in some barriers for analyzing species distribution patterns (Cobb et al., 2019; Elith & Leathwick, 2009; Gomes et al., 2018), the spatial and temporal data from these museum specimens can also inform evolutionary studies. For example, Spalink et al. (2016) used museum specimen occurrence data to quantify geographic distributions and ecological niches in North American sedges. They combined these data with a phylogeny and estimated speciation rates to show that geographic range changes influence both reproductive isolation and the evolution of niche space. Broad comparative approaches like this in insects and plants have also facilitated understanding evolutionary outcomes due to range shifts caused by both global climate change and urbanization (Kharouba, Lewthwaite, Guralnick, Kerr, & Vellend, 2018; Lahr, Dunn, & Frank, 2018; Meineke, Davis, Davis, & Davies, 2018).

Museum specimen occurrence records are important pieces of data, but when combined with supplementary information (e.g., photographic species records, land cover, or aerial photographs), such datasets can provide unique insights into the scale and mechanisms of species range shifts and habitat changes, including in urban areas. For example, using a combination of museum specimen and citizen science‐generated bird occurrence data, Battey (2019) showed that recent range expansions of Anna's Hummingbird in western North America are driven by ecological release associated with nectar from landscaped nonnative plants. Shultz, Tingley, and Bowie (2012)—using species records archived in museum field notes, records from a prior publication, and modern point count data—demonstrated community‐composition turnover in birds associated with long‐term increases in urbanization. In bumblebees, Macphail, Richardson, and Colla (2019) were able to demonstrate not only changes in species distribution, but also quantified extinction risk using a combination of museum specimens, general citizen science observations, and targeted surveys within the historic range of these bees. Museum records also serve as the vouchers, types, and physical evidence necessary for tracing historical and new introductions of invasive species (Puckett et al., 2016; Vendetti, Lee, LaFollette, & Citizen Science contributors to SLIME and BioSCAN., 2018) and for documenting new species discoveries in urban settings (Brown & Hartop, 2017; Hartop, Brown, & Disney, 2015, 2016). The value of museum records for documenting species presence in space and time will likely increase as more collections become widely available on digital data‐sharing platforms like VertNet and iDigBio (Constable et al., 2010; Nelson & Ellis, 2018).

### 2.3 Museum specimens are repositories of genetic material

Museum specimens are invaluable archives of genetic and genomic data, allowing studies of the genes and molecular mechanisms affected by habitat alteration and urbanization. Many natural history museums began developing dedicated tissue collections in the late 1970s, and some have tissue samples from much earlier (Sheldon, 2001). Importantly, data generated from genomic resource collections can be complemented with genomic data from museum material that was never intended for molecular analyses. New techniques have increased the efficiency of extracting genomic data from a wide variety of traditional museum specimens, including dried study specimen toe pads (Linck, Hanna, Sellas, & Dumbacher, 2017; McCormack, Tsai, & Faircloth, 2015; Tsai, Schedl, Maley, & McCormack, 2019), skin samples (Bi et al., 2013), eggs (Lee & Prys‐Jones, 2008), bones (Rowe et al., 2011), teeth (Pichler, Dalebout, & Baker, 2001), mollusk shells (Andree & López, 2013; Der Sarkissian et al., 2017), formalin‐fixed fluid‐preserved specimens (Derkarabetian, Benavides, & Giribet, 2019; Hykin, Bi, & McGuire, 2015; Ruane & Austin, 2017), and dried whole insect specimens (Kanda, Pflug, Sproul, Dasenko, & Maddison, 2015). While DNA from these sources tends to be highly fragmented and degraded, these approaches are promising and dramatically expand the potential temporal extent of studies and the number of samples available to analyze for urban molecular evolution research. Furthermore, as the methods to map phenotype to genotype improve, those working with museum specimens may be able to study allelic change over time based only on phenotype, and without molecular material. While this has yet to be applied to urban‐living taxa, Des Roches, Bell, and Palkovacs (2019) used lateral plate number in stickleback fish from museum collections as a proxy for alleles, and documented allele frequency shifts over time.

The maintenance and growth of genomic resource collections in museums worldwide and the ability to extract molecular data from traditional museum specimens has enhanced and increased the resources useful for understanding urban‐living taxa and their biotic responses to urbanization. For example, these data may be used to identify exotic species (Grimaldi et al., 2015), understand how urbanization influences contemporary population structure and connectivity (Richmond et al., 2017), and detect temporal trends in demography and genomic diversity (Ochoa, Gasca, Ceballos, & Eguiarte, 2012). Molecular techniques and approaches using museum specimens for nonurban studies can also provide insight and direction for future urban evolution studies. For example, exon capture of Eurasian rabbits collected in the United Kingdom, France, and Australia before and after the myxoma virus was released in the 1950s showed the evolution of resistance via polygenic, parallel selection (Alves et al., 2019). Targeted exon scans across museum mammal specimens from museum collections made a century apart revealed signatures of selection associated with changing climate (Bi et al., 2019), and RNA extracted from high‐quality genomic resource collections found transcriptomic plasticity across habitat gradients (Cheviron, Whitehead, & Brumfield, 2008). Opportunities to study urban evolution will continue to expand with the growth of genomic resource collections and with efforts to archive diverse types of tissues appropriate for DNA and RNA studies.

### 2.4 Opportunities to apply the extended specimen to studies of urban evolution

Technological advances, more digital resources, and increasing interests in varied aspects of species’ biology are yielding diverse data types associated with individual museum specimens (Schindel & Cook, 2018; Webster, 2017). Some of these data types have traditionally been referred to as derivative or ancillary collections. More recently, leveraging new technologies to both diversify the data associated with a museum specimen and increase accessibility to those data via digital archives has given rise to the concept of the extended specimen (Lendemer et al., 2020; Schindel & Cook, 2018; Webster, 2017). Aspects of the extended specimen could include tissue samples, stomach contents, fecal samples, parasites, histology slides, and digital media including audio recordings, photographs, videos of the organism and/or its environment, X‐rays, and CT scans. In the context of urbanization, studies could use gut contents to examine changes in dietary patterns, ecto‐ or endoparasite loads, or timing of reproduction over the course of habitat modification, as well as examine evolutionary mechanisms underpinning such changes. For example, Herrel et al. (2008) studied diet, head morphology, and soft tissue anatomy in native and introduced wall lizards and found that increased herbivory in an introduced island population was linked to changes in head morphology and the evolution of a cecum. Evidence of ecological changes associated with urbanization can be inferred from evidence of species interactions. For example, Kleijn and Raemakers (2008) used changes in pollen loads on bee specimens to highlight that a loss in bumblebee species was associated with the loss of their host plants, specifically in urban environments. Signs of herbivory or disease can also be gleaned from plant specimens, which can indicate changing species interactions in urban areas (Meineke & Davies, 2018).

With strategic sampling, urban‐collected specimens can also be used for studying stable isotopes, evidence of disease and/or parasites, and an organism's microbiome (Harmon, Littlewood, & Wood, 2019; Missagia, Patterson, & Perini, 2019; Rocque, Winker, & Zink, 2005). Museum specimens can also be tested for environmental contaminants such as synthetic organic compounds and heavy metals (Rocque et al., 2005). For example, examination of bird eggs collected across decades found an increase in chlorinated hydrocarbons in egg residue as well as eggshell thinning immediately after the introduction of the insecticide DDT while shell thinning was dramatically reducing reproductive success in fish‐eating birds (Hickey & Anderson, 1968). More recently, DuBay and Fuldner (2017) used bird specimens collected over 135 years in Illinois, USA, to document how the dirtiness of bird plumage reflected environmental air quality policies.

The diverse data types that make up the extended specimen showcase the many creative research uses of museum specimens. Over the centuries of specimen collection, the original collectors could not have imagined the many creative ways their specimens could be and have been used. In recent decades, the advent and expansion of genetic and genomic technologies, stable isotopes, and other techniques (Schmitt et al., 2018) allow novel types of research. Similarly, contemporary specimen‐based researchers cannot predict the diverse future uses of the specimens they archive.

## 3 USE AND DEPOSITION OF MUSEUM SPECIMENS IN PUBLISHED LITERATURE

We hypothesize that museum specimens currently play a minor role in studies of urban evolution, although they have been used in some key studies (examples above). To test this hypothesis, we conducted a literature review of broadly impactful and taxon‐specific journals (Methods S1; Table S1) to quantify both the proportion of published urban evolution studies that used museum specimens and the proportion that reported the deposition of specimens into museums or other appropriate collection repositories. We restricted our quantitative review and analyses to nonfossil terrestrial organisms, while acknowledging that marine environments are also impacted by urbanization and other anthropogenic influences (e.g., Reid et al., 2016; Therkildsen et al., 2019; Whitehead, Clark, Reid, Hahn, & Nacci, 2017). Likewise, we are keenly aware that anthropological, archeological, and paleontological data are necessary and important for reconstructing habitats, tracking species range shifts, and understanding trait evolution through time (Allmon, 2005; Davis & Pyenson, 2007; Harnik, 2009; Rick & Lockwood, 2013), but are beyond the scope of this paper. Also, in various examples included herein, herbaria are considered natural history museums.

**FIGURE 2:**
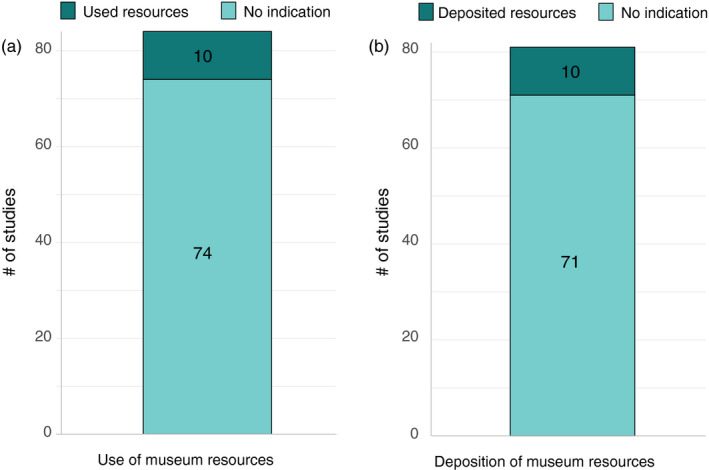
The number of urban evolution studies that (a) indicated that they used museum resources, and (b) indicated that they deposited resources in museums or appropriate repositories. Three studies did not create a new resource and were not included in (b)

We searched for urban evolution‐focused journal articles published between 2009 and 2019 in 18 total journals, some broadly impactful and some taxon‐specific. Although far from exhaustive, this review provides a representative sample for surveying museum specimen use in urban evolution studies. Out of 84 urban evolution studies, only 12% indicated using museum specimens. Notably, in 96% of studies, specimens were collected or some other archivable resource was produced (e.g., photographs, bird songs, or tissue samples); although these studies could have deposited collected materials into an appropriate museum collection or repository, only 12% stated that they actually did so (Figure 2; Table S1). In our opinion, these results indicate that museum specimens are not being as widely used as they could be for urban evolution research and collected material is not being adequately archived for the future. It is possible that some studies in our review used museum specimens but did not cite them as coming from museums (i.e., our results underestimate museum specimen use in urban evolution studies) or that collected materials were deposited in museums but that was not stated in the publication (i.e., our results underestimate the frequency of archiving collected specimens or other materials), but such improper reporting is also problematic. Indeed, we found that several studies indicated they used museum specimens but did not properly credit the collection or include the cataloged museum specimens used (for recommendations, see Section 5.1).

As mentioned above, the preponderance of studies we reviewed (96%) did create museum resources (e.g., collected specimens, photographs, or recorded sounds), which is encouraging. While some of these samples may have been deposited into museums and not reported, or some researchers may be in the process of depositing samples, we suspect that the vast majority of collected material was discarded or is stored in an individual or laboratory's collection where it is not openly available to other researchers. Regardless, if no indication of specimen deposition is made in the previously published results, researchers will not know to look for these specimens in museum archives so the utility of these specimens as archives of primary data is greatly reduced. We also acknowledge that some biologists may have had difficulty finding, or were not aware of, an appropriate repository that would accept specimens. We hope that by communicating to these biologists the value of their specimens as vouchers in museums, many will develop partnerships with museum collection professionals and will choose to deposit at least some of the resources they have generated. Such partnerships are essential to the sustainability and relevance of museum collections, as limited funding, undersized staff, required collection permits, and other hurdles make it impossible for museums alone to adequately document biodiversity in urban areas (or elsewhere).

## 4 TEMPORAL AND SPATIAL PATTERNS OF VERTEBRATE SPECIMEN DEPOSITION

**FIGURE 3:**
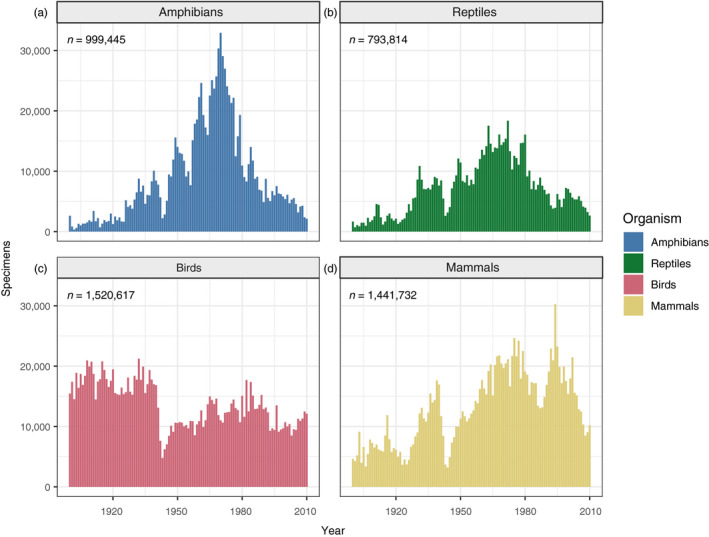
The number of specimens deposited in the United States as reported by VertNet per year from 1990 to 2009, for (a) amphibians, (b) reptiles, (c) birds, and (d) mammals. Total specimen numbers for each taxon included in the analysis are indicated in the upper left of each plot

The studies of evolution in urban environments, highlighted above, are only possible if the specimens exist to be studied. The extent of museum holdings for plants (Daru et al., 2018; Meineke, Davis, et al., 2018) and invertebrates (Cobb et al., 2019; Winston, 2007) has been quantified previously, but similar assessments of vertebrate specimens are lacking. We analyzed VertNet records with localities in the United States (Figure 3) and worldwide (Figure S1) of amphibians (Bloom, 2018a), reptiles (Bloom, 2018d), birds (Bloom, 2018b), and mammals (Bloom, 2018c). First, we examined patterns of specimen deposition through time by counting the number of specimens deposited per year for each group, between 1900 and 2009. We excluded 2010–present because not all museum catalogs are up to date online. In the United States, one consistent pattern emerged across all groups: a sizable decrease in specimen deposition during World War II in the early 1940s (Figure 3). Amphibian and reptile specimen deposition peaked in the late 1960s, followed by a marked decrease from 1970 to the present. Birds showed remarkably stable patterns of specimen deposition from 1900 to World War II, and stable, but decreased deposition afterward. We speculate that the decreased collecting intensity of birds was due to an increased difficulty in acquiring permits for bird collecting, possibly following the establishment of the U.S. Fish and Wildlife Service and the passage of the Convention on Nature Protection and Wildlife Preservation in the Western Hemisphere, both of which occurred in 1940 (U.S. Fish & Wildlife Service,n.d.). In the early 2000s, a steady deposition of bird specimens into museums may be explained by a partial shift in strategy for specimen acquisition (i.e., salvage), which we discuss in the following paragraph. Like reptiles and amphibians, mammal specimen deposition increased through time with a peak in the late 1960s. However, the decline in mammal specimen deposition in the United States was much less pronounced compared to amphibians and reptiles. Interestingly, mammal specimen deposition trends worldwide were more similar to those for amphibians and reptiles (Figure S1), with a much larger peak in the 1960s and 1970s, potentially due to a decrease in large, international collecting expeditions.

**FIGURE 4:**
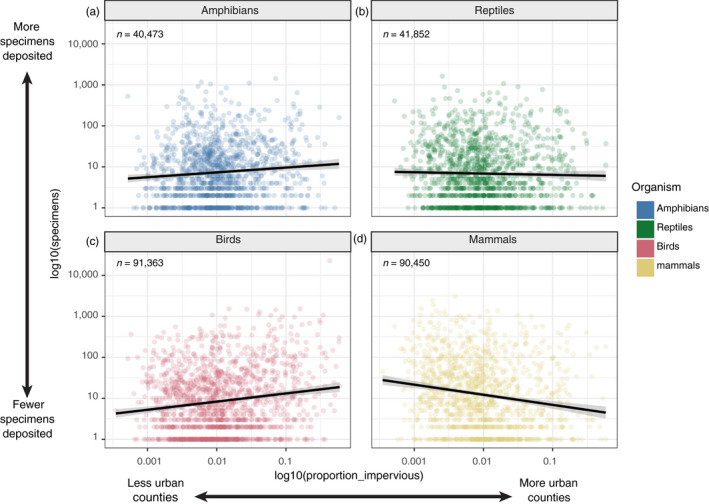
The number of (a) amphibian, (b) reptile, (c) bird, and (d) mammal specimens deposited between 2000 and 2009 in the United States plotted against the proportion of impervious surface (as a proxy for urbanization) for counties in the United States from which at least one specimen was deposited. Both measurements have been log‐transformed to facilitate visualization, and the relationship is shown with a linear model fit to these log‐transformed data. Total specimen numbers for each taxon included in the analysis are indicated in the upper left of each plot

We next sought to analyze recent (2000–2009) specimen deposition patterns in rural versus urban/developed areas. For each county in the contiguous United States, we counted the number of specimens collected across our four vertebrate groups of interest and compared these numbers to the percent impervious surface for each county, as a proxy for degree of urbanization. Detailed methods for obtaining these estimates and associated statistics are in Methods S1. Our results show a strong positive relationship between urbanization and specimen deposition rates for birds (ϐ = 0.39, *SE* = 0.03, *p* < .0001; Figure 4c), a moderately positive relationship for amphibians (ϐ = 0.11, *SE* = 0.03, *p* = .001; Figure 4a), a negative relationship for mammals (ϐ = −0.19, *SE* = 0.03, *p* < .0001; Figure 4d), and no relationship for reptiles (ϐ = −0.01, *SE* = 0.03, *p* = .674; Figure 4b). We hypothesize that the different results across groups may be related to methods of specimen acquisition. In birds, many museums currently acquire a substantial proportion of new specimens via salvage programs and wildlife rehabilitators, both of which are more common in urban areas, and in close proximity to natural history museums. This phenomenon is exemplified by the outlier data point at the top right of Figure 4c; it indicates Cook County, Illinois, which contains the city of Chicago and the Field Museum of Natural History, which acquired more than 22,000 bird specimens deposited between 2000 and 2009. The Field Museum has an active salvage program that collects, in particular, birds killed by building strikes during migration (Weeks et al., 2020). The negative correlation between mammal specimen deposition and level of urbanization may be driven by researchers collecting in natural areas rather than acquiring specimens through salvage or by actively collecting in urban areas. Finally, the weak relationships between urbanization and specimen deposition in amphibians and the lack of a trend in reptiles may be explained by the coarseness of our analysis: County, as a category, could be too large an area to capture the relationship between these animals and the habitat from which they were collected. That is, for reptiles and amphibians, researchers may be more likely to collect near their homes and workplaces, or collect from sites easily accessible by roads, a pattern also observed in plants (Daru et al., 2018). Spear, Pauly, and Kaiser (2017) examined geographic biases around specific collection localities in Southern California for four species of reptiles and amphibians and found a bias against specimen collecting in urban areas, despite the overall large urban footprint of the area studied.

**FIGURE 5:**
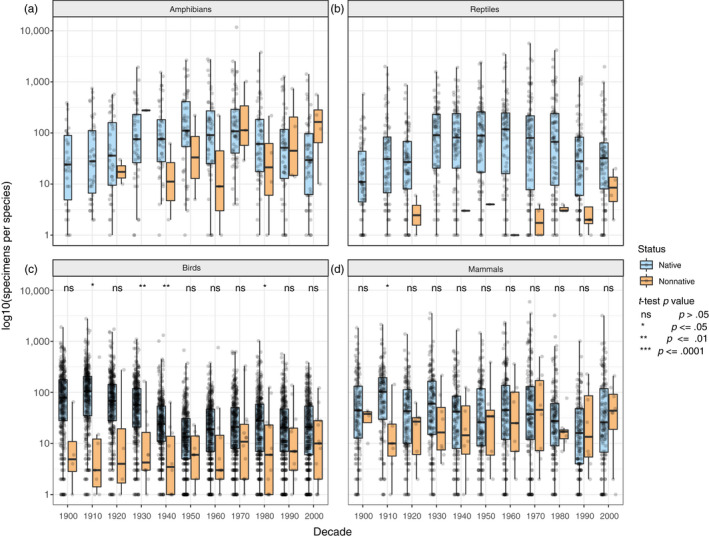
The number of museum specimens deposited per native and non‐native species each decade from 1990 to 2009 in California for (a) amphibians, (b) reptiles, (c) birds, and (d) mammals. Numbers have been log‐transformed for easier interpretation, and boxplots and raw data points are included. For birds and mammals, significance of *t* tests between the means of native and non‐native species is shown at the top of each panel. There were insufficient species sample sizes for amphibians and reptiles to conduct statistical tests

Non‐native species collected from urban areas are invaluable to understanding how organisms evolve in urban environments (Dlugosch & Parker, 2008; Phillips & Shine, 2004). However, we anticipated a bias against collecting non‐native species either because collectors are more inclined to value native specimens or because the acquisition of specimens in some organismal groups is restricted to native species (e.g., wildlife rehabilitators may not accept nonnative species). To understand specimen deposition patterns for native and nonnative species, we analyzed differences between native and non‐native terrestrial vertebrate species in California deposited in museums every decade from 1900 to 2009. We chose California as a representative subset of VertNet data because it has diverse environments and several major urban areas, yet is small enough to score each species appropriately. For each species, we summed the number of specimens collected per decade (details in Methods S1) then categorized each as “native” or “non‐native” and compared the average number of specimens per non‐native species to the average number of specimens per native species for each decade with *t* tests. Amphibians and reptiles did not have enough catalogued non‐native species for statistical comparison (Figure 5a,b). For birds, there were four decades with significantly fewer specimens collected per non‐native species (1910, 1930, 1940, 1980; *p* < .05; Figure 5c), and for mammals, there was one decade with significantly fewer specimens per non‐native species (1910; *p* < .05; Figure 5d). Although all five comparisons with a significant difference show non‐native species being under‐collected relative to native species, these comparisons are in the earlier decades. Overall, our analyses show increasing numbers of non‐native specimens deposited through time. This appears especially true for amphibians, in which the median number of non‐native specimens per species deposited was over 5‐fold greater than median numbers of native specimens per species in the last decade (Table S2). Reptiles showed a modest trend towards an increase in non‐native specimen deposition over time, but fewer specimens per species than most native species. Birds showed a slight increase in the number of non‐native specimens deposited through time, but the decrease in significant differences between native and non‐native species through time may reflect the deposition of fewer native specimens rather than more non‐native specimens. Mammals showed the most similarity between the number of native and non‐native specimens deposited through time. Overall, while results show increasing collecting and deposition of non‐native specimens for these vertebrate groups, we contend that non‐native specimen deposition should be much higher given the relaxed permitting required to collect most of these taxa and decreased conservation concern (e.g., some are harmful to native species). Also, while the number of non‐native specimens being deposited may be similar to the number of native specimens being deposited for some species, there are many non‐native species without any specimens deposited. For example, in the last decade 13 species of nonnative birds collected in California had at least one specimen deposited in a museum collection, but during that time, there were 38 non‐native bird species known in the state (Garrett, 2018). Thus, in this case (and likely others), non‐native populations are not accurately represented in museum collections.

Overall, our analyses of VertNet specimen data indicate that while specimen deposition could improve, many museum specimens exist for the study of evolution in urban environments, suggesting that they are likely underutilized (Figure 2). Similar results were found for plants (Daru et al., 2018; Meineke, Davis, et al., 2018) and invertebrates (Cobb et al., 2019). While specific collecting may be necessary to augment existing samples, museum specimens could provide a prior baseline for comparison, or reduce the number of individuals that need to be collected to complete a study. Beyond recognizing collections as a resource, changes to practices and attitudes toward museums are necessary throughout biological science—by numerous individuals from researchers to journal editors and funding agencies—to ensure specimens will be available for the future. Below, we make recommendations to build and improve museum collections for urban evolution studies (Section 5).

## 5 RECOMMENDATIONS TO DIRECTLY ADDRESS THE OBSTACLES TO URBAN SPECIMEN COLLECTION AND USE OF COLLECTIONS

Archiving specimens in museums presents an unparalleled opportunity to build resources that document biodiversity in changing environments, such as areas experiencing rapid urbanization. These resources serve many purposes, but their primary value is in two areas. First, archiving specimens or samples that are used in published research preserves them in the event that studies need to be re‐visited, facilitating the repeatability that is core to scientific research. Second, it captures organismal data from a snapshot of a particular place and time, providing records that are impossible to retroactively create. Our findings of urban and nonnative specimen deposition illustrate several points that can guide efforts to build collections of urban animals. For some taxa (e.g., mammals), there are disproportionate specimen collection efforts focused on nonurban areas (Figure 4). Although some taxa (e.g., birds) are well‐represented from some urban areas, these trends may be driven by intense efforts in relatively few urban settings (e.g., the Field Museum in Cook County, Illinois, USA, or the Natural History Museum of Los Angeles County in California, USA). Additionally, many of the birds that are salvaged from window strikes are migrating species, and not breeding “urban” species. This haphazard sampling creates biases in specimen availability. Therefore, it is important to deposit population‐level samples of high‐quality material (e.g., cryogenically frozen RNA quality tissue samples) from native and non‐native species to enrich and improve these snapshots of biodiversity for future scientists.

Below we make recommendations for improving archiving and access to samples and specimens in appropriate repositories. We consider appropriate repositories to be museums or other collections with dedicated curatorial and collections management staff and an institutional commitment to digitize specimen records and make them available to data aggregators like VertNet, GBIF, and iDigBio. General best practice recommendations for specimen deposition have been made for some taxa: arthropods (Turney, Cameron, Cloutier, & Buddle, 2015), plants, (Meineke, Davis, et al., 2018), and mammals (McLean et al., 2016). Here, we primarily focus on topics relevant to urban studies and the analyses we have conducted. These are not absolute guidelines, and responsible parties should obviously use best judgment, with the overall aim of increasing availability of biological data. Successfully implementing this approach to archiving specimens and samples in museums cannot come from a single group, such as museum scientists, but requires a paradigm shift from many different stakeholders.

### 5.1 Recommendations for researchers

As indicated by our analysis, numerous published studies with an urban focus did not deposit collected specimens or failed to indicate whether collected specimens and/or samples were deposited in museum collections (Figure 2). Not only does this curtail the reproducibility of research, if specimens are not deposited, it limits the resources available to future scientists. Researchers may be concerned that depositing specimens could limit their own use of them or allow them to be “scooped” by others. However, museums can place restrictions on deposited material for a set period of time before it becomes available for general research use (similar to molecular data deposited to the United States’ National Center for Biological Innovation or NCBI). Contemporary researchers should keep in mind that the future research conducted by urban evolutionary biologists will be determined, in part, by the museum specimen resources created by today's researchers. To create robust urban‐collected specimen resources in natural history museums, researchers should:

● Deposit some to all of their collected specimens and/or derivative material in appropriate collections or repositories to ensure that their science is reproducible and to build the capacity and resources for future studies (Schilthuizen, Vairappan, Slade, Mann, & Miller, 2015; Ward, Leschen, & Buckley, 2015).
● Provide the catalog numbers of examined specimens in published research methods or supplementary materials to facilitate reference to specific specimens.
● Seek advice from museum personnel before specimen collection is begun to ensure collection of all necessary data, and, if applicable, discuss financial needs to house and curate deposited specimens.
● For the museums that accepted collected material and/or provided collections for examination, acknowledge the museums and collections appropriately in published research.

### 5.2 Recommendations for museum professionals

For many taxonomic groups, specimens have been historically collected by trained systematists employed at natural history museums. Current trends indicate that fewer undergraduate students are being trained in natural history and systematic methodologies. Museums are uniquely positioned to offer training opportunities in systematics and museum studies, through undergraduate programs at museums within universities (e.g., the program in the Museum of Vertebrate Zoology at UC Berkeley; Hiller et al., 2017) and through partnerships between universities and stand‐alone museums (e.g., an internship in museum studies at the Natural History Museum of Los Angeles County offered by Glendale Community College; Vendetti & Gago, 2018). Taxonomic experts at natural history museums can also address a dwindling pipeline of natural historians and taxonomists (Tewksbury et al., 2014) by leading taxon‐specific courses in identifying and preparing museum specimens (e.g., fly school (https://dipteracourse.com/, last accessed February 5, 2020) or ant course (https://www.calacademy.org/scientists/ant‐course, last accessed February 5, 2020)).

Museum scientists are likely contributing to some of the trends we see in urban specimen deposition. Curators and collections managers frequently accept or actively collect specimens from “natural” or “wild” areas; however, museum collections may not accept non‐native specimens or common urban species. Many museum institutions are severely limited in capacity and financial support for specimens, so it may not be possible to accept all potential samples and specimens. We urge museums and collections professionals to consider the value of urban and non‐native specimens (which can also be expensive and difficult to collect due to the diverse mosaic of property ownership) and accept, at minimum, a subset of collected specimens as representatives whenever possible. Museum personnel should consider the following suggestions to create robust urban‐collected specimen resources in museums:

● For deposition requests, accept, at minimum, a subset of urban‐collected specimens, especially those included in published research.
● Broadly communicate (e.g., at scientific meetings, through institutional websites, appropriate listservs, and relevant research groups) the collection's willingness to accept specimens and guidelines for acceptance.
● Publish contact information for use by researchers seeking to deposit specimens and make protocols available for specimen documentation and/or initial preparation necessary to make specimens suitable for deposition.
● When appropriate, develop salvage programs for rare or hard to collect urban‐living taxa. Consider that volunteers and appropriate protocols to save salvaged organisms can greatly increase the intake of such specimens.
● Georeference and digitize specimen records to increase the digital accessibility and utility of data from urban‐collected specimens.
● Enhance the potential use of specimens by building derivative collections, especially of tissues, parasites, and gut contents.
● Resample localities and populations consistently over time to build a temporal series of urban‐living species (including recently arrived nonnative species) that can be used for urban evolutionary studies.
● Organize training opportunities for students and volunteers in systematics, taxonomy, and/or best practices in specimen preparation and curation.
● Upload digital specimen data to data aggregators, and ensure that the data aggregator correctly indicates when genetic resources or other derivative material are available.

### 5.3 Recommendations for scientific journal editors, funding agencies, and university administrators

Museum collections need both financial and institutional support to process and accommodate urban‐collected specimens. Needs include personnel time for processing and data entry, consultation with and contracting of taxonomic experts, institutional overhead and facilities costs, and specimen housing and supplies costs (Baker, Bradley, & Bradley, 2014; Bradley, Bradley, Garner, & Baker, 2014). Some of these are one‐time expenses, while others continue throughout the specimen or sample's “lifetime” in a collection. There are mechanisms to increase support for these efforts both directly and indirectly. First, museums need increased funding from both public and private institutions. Funding agencies and private entities should create opportunities to support continued efforts to maintain current operations in addition to support for new efforts. Second, direct support for the curation of specimens is often omitted from grant budgets but should be included to offset costs for specimen curation (e.g., mammal curation costs, see Bradley et al. (2014)). Third, by requiring that specimens be appropriately deposited and cited as part of the publication and funding processes, museums will gain both valuable resources and recognition for the important role they play in science, raising their visibility and increasing support. These aims are comparable to the requirements of journals and funding agencies that data and analyses be deposited in publicly available archives such as GenBank and Dryad.

● Journal editors should require that, in all but exceptional cases, the author(s) of submitted manuscripts that include information from collected specimens, deposit at a minimum, representative samples of collected and archivable resource(s) in an appropriate repository, before their manuscript can be considered for review.
● Journals should require that specimen or catalog numbers and the collection and museum from which they came or wherein they were deposited be reported in the publication.
● Funding agencies should require that at a minimum, representative specimens or samples collected using grant funding be deposited in an appropriate repository.
● Funding agencies should encourage the inclusion of costs related to specimen loans and specimen deposition in research grant budgets.
● Funding agencies should offer funding support to museums directly for the acquisition and maintenance of specimens acquired through public funding.
● University/college administrators who oversee institutional museums and/or biological collections should allocate financial support for collection maintenance, growth, and personnel.
● University/college administrators who oversee biology faculty that actively collect biological specimens and/or samples should consider that faculty member's commitment to the deposition of museum resources should be included in any promotion policies regarding data sharing and reproducibility.

### 5.4 Recommendations for permitting agencies and animal care and use committees

Agencies and committees that regulate the use of biological specimens have the power and opportunity to influence specimen deposition of urban‐living taxa. McLean et al. (2016) found that less than half of U.S. state wildlife agencies require that specimens collected by permittees be deposited in a museum collection. Requiring permit applicants to deposit at least some specimens in appropriate collections will increase specimen deposition rates and ensure that data from animals that are handled or euthanized are maximized. Permits are also necessary for acquiring salvage material (animals found dead) for many vertebrate groups. Individuals with appropriate permits can accept salvaged animals legally with permits, but any members of the public transporting salvaged animals do so illegally, limiting the ability of museums to communicate the need and abilities for these types of specimens. Legalizing the transportation of salvaged animals to museums would allow museums to advertise broadly their ability to accept specimens, increasing the availability of specimens from urban areas.

Non‐native species, which may be common in urban areas, sometimes do not require permits for collecting or other scientific sampling. However, most research on vertebrates, including non‐native species, is subject to oversight by Institutional Animal Care and Use Committees (IACUCs). One goal of these committees is to minimize the number of animals that are euthanized in the name of science (“Public Health Service Policy on Humane Care & Use of Laboratory Animals”, 2015). By requiring the deposition of urban‐collected specimens and samples in an appropriate repository, wildlife agencies, IACUCs, and other relevant stakeholders contribute to the growth of collections useful to future researchers who may then choose to sacrifice fewer animals because of these available specimens.

● Permitting agencies should require that some proportion of biological samples or specimens collected under scientific collecting permits be deposited in an appropriate repository.
● Permits to deposit salvaged specimens in museum collections should be widely approved and have mechanisms that allow members of the public to legally contribute salvaged specimens to appropriate repositories.
● Institutional Animal Care and Use Committees (IACUCs) should only approve protocols that include a plan to deposit sacrificed animals or sampled tissue in an appropriate repository.

### 5.5 Recommendations for taxon‐focused professional societies

Some professional societies publish guidelines on the use of wild animals in research and could encourage the deposition of specimens and biological samples into museum repositories. The Guidelines for Use of Live Amphibians and Reptiles in Field and Laboratory Research (Beaupre, Jacobson, Lillywhite, & Zamudio, 2004) and Guidelines for the Use of Fishes in Research (Jenkins et al., 2014) explicitly mention collecting specimens for museums. Guidelines for the Use of Wild Birds in Research (Fair, Paul, & Jones, 2010) and Guidelines of the American Society of Mammalogists for the Use of Wild Mammals in Research and Education (Sikes & Animal Care and Use Committee of the American Society of Mammalogists, 2016) both advocate archiving specimens in museums. Most guidelines make reference to depositing whole specimens; however, recommendations could be expanded to include depositing other biological samples in a museum collection or repository (as discussed in Section 2.4). As many animal care and use committees refer to taxon‐specific guidelines mentioned above, adding an explicit recommendation about specimen deposition would contribute to building museum collections with these valuable resources.

● Taxon‐focused professional societies should include depositing specimens in museums as part of their guidelines for the use of wild animals in research.
● Encourage the examination of museum collections for research purposes and advocate for the deposition of collected specimens and biological samples into museum/collection repositories in electronically or otherwise published society literature.
● Include depositing collected whole specimens as well as other biological samples into museum/collection repositories as part of guidelines for the use of wild animals in research.

## 6 CONCLUSIONS

Urbanization drives rapid evolutionary changes by creating new, dynamic environments, and facilitating novel interactions among species. Natural history museums provide both the historical time series of collections needed to document that change and the facilities required to ensure those resources are available for generations of scientists to come. Nonetheless, we demonstrate that for some vertebrate groups, museum collections are currently underutilized, and, most importantly, that specimens and samples from urban environments are being collected but not adequately deposited in museums. Creative uses of specimens and the derivative material collected with them have produced invaluable insights into how species are changing on historic timescales. As technologies develop that create novel research methods, these specimens will only become more valuable. Collections are an irreplaceable resource that investigators across disciplines should be contributing to and acknowledging. With proper acknowledgement, use, growth, and support, natural history collections will be invaluable resources for understanding urban‐living taxa of the present and future.

## CONFLICT OF INTEREST

None declared.

## Supporting information

Fig S1

Table S1

Table S2

Method S1

## Data Availability

Data for this study come from publicly‐available datasets, or are presented as a supplemental table.
