# Supplementary material for: Natural history collections are critical resources for contemporary and future studies of urban evolution
Source: Evol Appl. 2020 Jul 2;14(1):233–47. doi: 10.1111/eva.13045 (PMC7819571; doi:10.1111/eva.13045)
Supplement: Supplementary file 1 — Fig S1 [file EVA-14-233-s001.pdf]

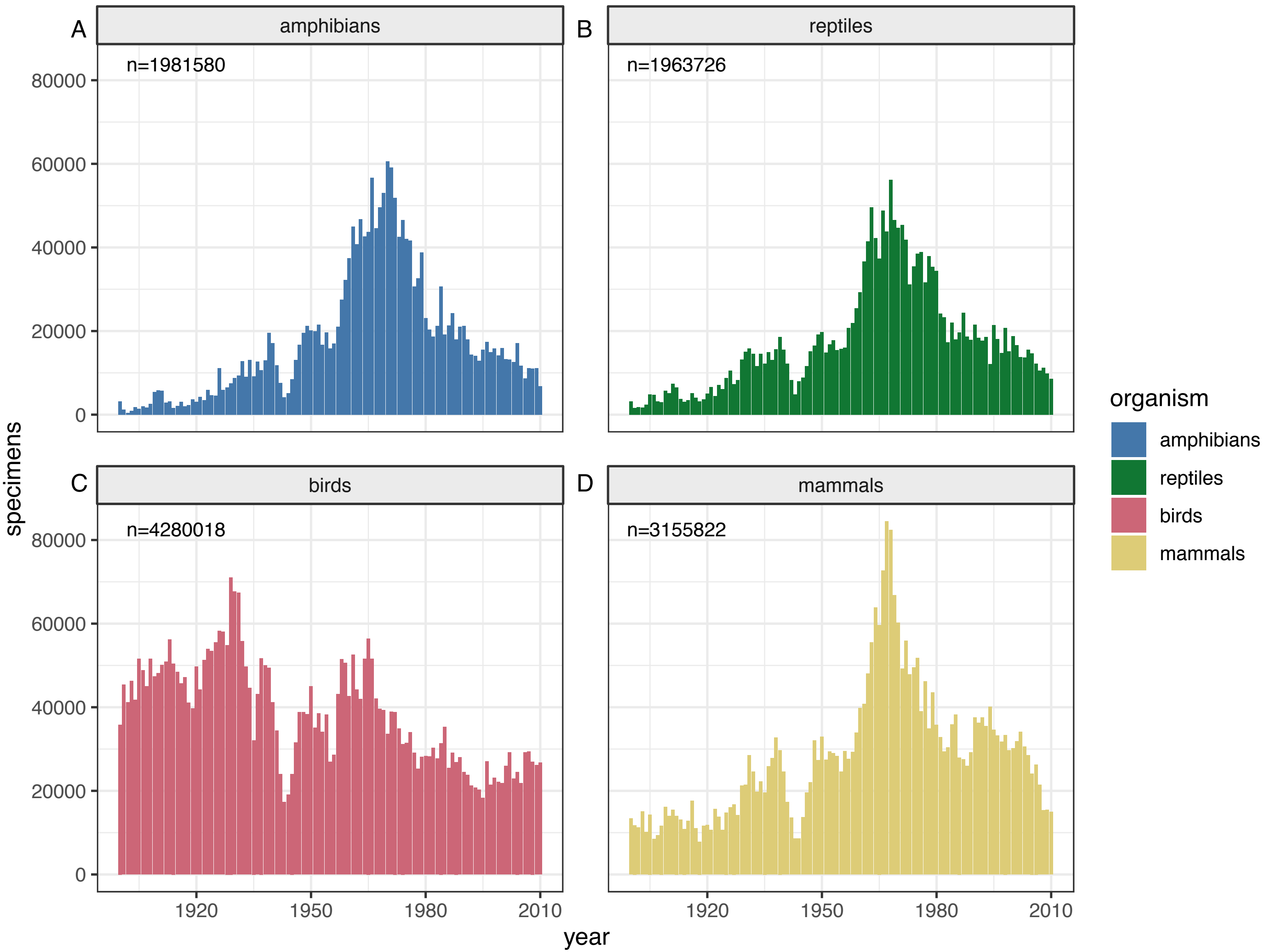

Supplemental Figure 1. The number of specimens deposited worldwide in VertNet per year for A) amphibians, B) reptiles, C) birds, and D) mammals.
