# Supplementary material for: Natural history collections are critical resources for contemporary and future studies of urban evolution
Source: Evol Appl. 2020 Jul 2;14(1):233–47. doi: 10.1111/eva.13045 (PMC7819571; doi:10.1111/eva.13045)
Supplement: Supplementary file 4 — Method S1 [file EVA-14-233-s004.pdf]

Supplemental Methods

Urban Evolution Literature Review

We conducted a literature review of a selection of broadly impactful and taxon-specific journals (Supplemental Table 1) to quantify both the proportion of studies that use museum specimens in their sampling design and the proportion of published studies that report the deposition of specimens in natural history collections. To do this, we conducted a search on the Web of Science database using the search terms “urban*” AND “evolution*”. We searched for papers within the following 18 journals: Science, Nature, Scientific Reports, PLOS One, Proceedings of the Royal Society B, Biological Journal of the Linnean Society, Evolutionary Applications, Molecular Ecology, Evolution, Journal of Urban Ecology, Environmental Toxicology and Chemistry, Journal of Mammalogy, Copeia, The Auk, Journal of Herpetology, Journal of Molluscan Studies, Annual Review of Entomology, and The American Journal of Botany. These journals were chosen to include representatives of broadly impactful journals, or journals that would reach a diverse audience (read by an audience from a broad taxonomic and/or subdiscipline background), and journals that were taxonomic-specific and included one journal for each major taxon. We limited the search to papers published between 2009 and 2019 to focus primarily on current practices of specimen use and deposition. The initial search results yielded 236 papers which were reduced to 84 papers after excluding reviews, projects solely focused on human or social changes, and projects which only relied on lab-reared organisms (Supplemental Table 1). We scored each study by whether they reported using museum specimens in their sampling (e.g., acknowledged a museum for specimens and/or provided specimen accession numbers; studies that could not have used specimens were marked as “not applicable”), whether they produced a resource that could be deposited in museums (voucher specimen, tissue sample, image, or recording), and whether they deposited any of these resources into a museum collection (any indication that some representative specimens were deposited).

Analyses of Specimen Deposition Data

We downloaded the 2017 VertNet snapshots for the four taxonomic groups: amphibians (Bloom, 2018a), reptiles (Bloom, 2018d), birds (Bloom, 2018b), and mammals (Bloom, 2018c). We excluded fossil data by filtering all records with "fossil" in the preparation field, and excluded all records that had an entry in any of the following fields, indicating that they were include a stratigraphic position: "earliestorloweststage", "earliesteonorlowesteonothem", "esarliestepochorlowestseries", or "earliesteraorlowesterathem", "earliestperiodorlowestsystem". We further refined the non-avian reptile data to remove any families with no extant members. For birds, we excluded specimens from the Cornell Lab of Ornithology, given the large number of images and sounds that are part of the collection from the Macaulay Library. While these are important records, we wanted to focus our analyses on physical specimens given other digital records are a recent development and are limited in their utility.

To obtain the proportion impervious surface for each county, within Google Earth Engine (Gorelick et al., 2017), we subsampled the impervious surface map from the 2016 United States National Land Cover Database (Yang et al., 2018) using the 2016 US Census Bureau county map boundaries (United States Census Bureau, 2016). Within each county, we created a histogram of pixels by percent impervious surface, ranging from 0 to 100 and combined these data to create a single measurement of the proportion of impervious surface for each county. We explored the relationship between specimen deposition totals and urbanization using a generalized linear model (R package *glm.nb*; Venables & Ripley, 1994). To account for non-normally distributed residuals and overdispersion of the specimen count data, we performed a negative binomial regression treating the proportion of impervious surface in a county as a fixed effect and the number of specimens deposited as the response variable. Impervious surface proportion values were logit transformed prior to analyses to improve model fit (Warton & Hui, 2011).

To understand specimen deposition patterns for native and nonnative species, we analyzed differences between native and nonnative species deposition patterns in California. We chose California as its large size and diverse habitat should be broadly representative and it contains several large museums, but is small enough to score each species appropriately. First, we extracted all records with “California” in the “stateprovince” field from the four VertNet datasets used above. Next, we classified each species as “native” or “nonnative”, but excluded any species likely to be found only in zoos or ornamental gardens and marine mammals. We also excluded any species with fewer than five records in the entire dataset as these largely represented species that are not established in the area of interest. Next, for each species we counted the number of specimens deposited in museum collections in each decade, and compared the counts of native and nonnative species for each class of organisms for each decade. For each decade we excluded all species with no specimens collected so that we could compare rates of deposition between groups. There were not enough nonnative species of reptile or amphibian to statistically compare mean numbers of specimens collected per species between native and nonnative species, but for birds and mammals we compared average values with t-tests (counts of specimens and species per status category per decade available in Supplemental Tables 2-5).
